# Telemedicine-based adapted physical activity programs for pediatric oncology patients in active oncological care: a feasibility study

**DOI:** 10.3389/fonc.2025.1634626

**Published:** 2025-09-08

**Authors:** Linda Peli, Elisa Inselvini, Marta Cogliati, Eleonora Finazzi, Chiara Gorio, Alessia Angeloni, Joel Pollet, Richard F. Schumacher, Fulvio Porta, Filippo Bertoni, Massimiliano Gobbo

**Affiliations:** ^1^ Lega Italiana Lotta Tumori (LILT), Brescia, Italy; ^2^ Dipartimento di Scienze Cliniche e Sperimentali, Università degli Studi di Brescia, Brescia, Italy; ^3^ Azienda Socio Sanitaria Territoriale degli Spedali Civili di Brescia, Brescia, Italy; ^4^ IRCCS Fondazione Don Carlo Gnocchi, Milan, Italy

**Keywords:** hematology, telemedicine, exercise, rehabilitation, functional status, quality of life, survivorship, cancer

## Abstract

**Background:**

Survival rates for pediatric hematological malignancies have significantly improved over the last 50 years. However, children undergoing cancer treatment face long- term health challenges, including musculoskeletal impairments and reduced physical activity levels, which contribute to poorer quality of life (QoL) and elevated cardiovascular risks. Barriers to accessing traditional physical activity and rehabilitation programs necessitate innovative approaches like telemedicine-based interventions.

**Objective:**

The primary objective was to evaluate the feasibility and adherence of a telemedicine-integrated adapted physical activity (APA) program in pediatric oncology patients. Effectiveness, in terms of functional and psychosocial outcomes, was considered as an exploratory secondary endpoint. Although some statistically significant findings were observed, these results should be interpreted as supportive evidence and not as definitive proof of effectiveness.

**Methods:**

The study included children and adolescents (5–18 years) with onco-hematological conditions. Participants underwent baseline (Pre-APA) and six-month (post-APA) assessments using different functional tests and validated QoL evaluation tools (PedsQL^TM^). The intervention comprised personalized exercise regimens targeting strength, aerobic capacity, flexibility, and balance. Supervised training sessions were conducted both in-person and remotely via telemedicine solutions. Secondary outcomes included quadriceps muscle and hand- grip strength, balance, fatigue, and health-related QoL. Statistical analyses utilized mixed regression models to evaluate pre-post intervention changes and the impact of remote training.

**Results:**

Eighteen subjects (8 female, median age 12 years) were included. The APA program demonstrated high adherence (median 77.08%). Preliminary improvements were observed in QoL (PedsQL^TM^ child, p=0.007), fatigue (p=0.035), and functional performance (chair stand test, p=0.033). Regression analysis indicated a positive correlation between remote training frequency and QoL improvement (p=0.027, R²=0.235). Although strength measures showed no significant improvement, remote training influenced flexibility outcomes. Parental perceptions of fatigue and QoL also improved (p=0.049).

**Conclusion:**

Telemedicine-based APA programs are a feasible and effective approach for enhancing QoL and functional capacity in pediatric oncology patients. These preliminary findings support the integration of digital health solutions in long-term survivorship care, though larger studies are needed to confirm these results.

## Introduction

Over the past 50 years, survival rates for pediatric hematological malignancies have increased significantly, from nearly 0% to over 80%, thanks to advancements in science and improved therapeutic protocols (1).

Nevertheless, children with cancer and childhood cancer survivors face long-term health challenges resulting from hospitalization and treatment, including physical and psychosocial consequences. Among these, musculoskeletal impairments and reduced levels of physical activity are particularly common, significantly affecting their quality of life (QoL). Physical activity (PA) in children and adolescents with cancer is often hindered by the disease itself and the side effects of treatments. This reduction in PA contributes to additional short- and long-term complications. Indeed, the low levels of PA observed in children with cancer, both before and after treatment, are a key factor behind the elevated risk of developing cardiovascular diseases among survivors. Moreover, the exercise capacity of oncology patients is notably lower than that of their healthy peers (2, 3).

Research indicates that engaging in regular PA can mitigate some of these adverse effects, promoting better functional outcomes and overall well-being (4–6).

However, many oncology patients face barriers to accessing traditional services, including limited availability of specialized care for adapted physical activity (APA) programs, transportation challenges, and time constraints, making in-clinic exercise interventions often impractical, uncomfortable, and inconvenient (7).

Telemedicine has emerged as a promising solution to address these barriers by providing remote access to healthcare services, including rehabilitation and exercise interventions tailored for children in active oncological care and pediatric cancer survivors. The application of telehealth in this context enables the delivery of supervised exercise programs through various digital platforms, thereby enhancing accessibility and convenience for families, regardless of geographical constraints. Recent studies have demonstrated that telehealth-based exercise interventions are not only feasible but also effective in improving functional capacity and QoL among cancer survivors, highlighting their potential to maintain continuity of care (8–10).

As healthcare systems increasingly integrate digital health solutions into cancer management and rehabilitation, it becomes essential to explore their efficacy and optimize their implementation for pediatric populations. This study aims to add evidence on this topic by assessing the feasibility, adherence, and effectiveness of a mixed program of APA in pediatric oncology patients, including telehealth-based exercise interventions. Furthermore, implications for future research and clinical practice are discussed.

## Materials and methods

### Study design and ethical considerations

The study was a feasibility-focused, non-controlled clinical trial (NCCT) that integrates personalized physical activity programs and employs pre-post clinical evaluation, including psychometric assessments. The Institutional Ethics Committee approved the study protocol (protocol number: NP3323), and informed consent was obtained from parents or legal guardians, along with age-appropriate assent from participating children. The study adhered to the ethical principles outlined in the Declaration of Helsinki. The study protocol was registered in Open Science Framework (OSF): https://doi.org/10.17605/OSF.IO/Z5FPN

### Participants and settings

Eligible participants were children and adolescents aged 5 to 18 years who were diagnosed with oncological or hematological diseases and followed at the Pediatric Onco-Hematology Unit and the Pediatric Bone Marrow Transplant Centre (CTMO) of the Spedali Civili Hospital in Brescia, Italy.

Exclusion criteria were patients younger than 5 years or older than 18 years; patients with pre-existing motor disabilities that precluded participation in the APA program; and patients with cardiopathies presenting an ejection fraction of less than 40%.

### Intervention

The APA program was tailored to the individual’s physical capacity, clinical needs, and psychological condition. The goals were to improve muscle strength, aerobic capacity, flexibility, and balance while mitigating fatigue symptoms and enhancing quality of life.

The program included aerobic exercise (conducted below the anaerobic threshold to optimize endurance without inducing excessive fatigue), muscle strengthening (exercises targeting major muscle groups to counteract treatment-related muscle atrophy), and balance and coordination training (activities designed to improve neuromuscular control and proprioception).

All sessions were supervised by qualified professionals, including exercise medicine specialists and professionals with master’s degrees in motor sciences.

The contents, duration, and dose of the interventions are reported through the TIDieR checklist in **Supplementary Material 1**.

### Feasibility criteria

Feasibility in pilot and preparatory trials is defined as the extent to which the intervention and study procedures can be successfully delivered and implemented as planned. Following the recommendations of Lancaster et al. (2019) and Cramer (11), we focused on key feasibility domains related to the recruitment process, intervention adherence, and tolerability. Specifically, we prospectively defined the following core feasibility criteria:

Recruitment rate and recruitment duration — measuring the number of participants enrolled per month and the time taken to reach the target sample size, respectively, as accurate estimation of these parameters is essential for planning a larger trial.

Adherence to the telemedicine-integrated adapted physical activity program, assessed by the proportion of completed sessions relative to those scheduled, reflecting participant compliance and intervention practicality.

Tolerability, monitored through the recording of adverse events and participant retention, to ensure the safety and acceptability of the intervention within the pediatric oncology population.

Although NCCT studies are not designed to formally assess effectiveness, tracking these parameters aligns with best practices in feasibility research, providing essential data to support subsequent full-scale studies.

### Assessment

Each participant underwent a comprehensive assessment to establish their clinical condition before initiating the intervention (Pre-APA) and 6 months after the intervention (post-APA) to monitor progress and evaluate the efficacy of the intervention. Furthermore, the assessment paid special attention to psychological aspects and quality of life.

The outcomes evaluated are described as follows.

#### Muscle strength assessment

Isometric muscle strength assessment was conducted using various portable dynamometers, allowing for the identification of potential muscle asymmetries and strength deficits. The portable dynamometers MicroFET (Hoggan Scientific LLC, USA) for the deltoid muscle and MAF1 (Forza, OT Bioelettronica, Torino, Italy) for the quadriceps were used to measure isometric muscle strength in the major muscle groups. Amplifiers used during testing allowed differential measurements to identify muscular asymmetries or specific deficits. Their portability and ease of use make them ideal for school or clinical settings. The Handgrip Dynamometer (Deyard SN X000E6RQNH) was used to evaluate maximum isometric force production in the forearm flexors, metacarpophalangeal flexors, and thumb adductor muscles.

#### Functional tests

A combination of specialized functional tests was employed to evaluate balance, flexibility, and functional capacity, providing objective and reliable assessments of participants’ physical condition. The Chair Stand Test (12) evaluated lower limb strength, endurance, and balance by counting the number of sit-to-stand repetitions completed within a 30-second timeframe. The Chair Sit and Reach Test (13) measured lower back and hamstring flexibility by determining the distance between fingertips and toes when participants were seated in a standardized position.

#### Quality of life and fatigue assessment

Health-Related Quality of Life (HRQoL) was assessed using the Italian version of the PedsQL™ system (14), a validated, modular tool for measuring quality of life in pediatric populations. The system includes both child and parent-proxy reports, stratified into three age groups: 5–7, 8–12, and 13–17 years. The Pediatric Quality of Life Inventory was administered using the following modules: PedsQL™ Generic Core Scales (PedsQL™ GCS), which assess four domains: physical functioning (8 items), emotional functioning (5 items), social functioning (5 items), and school functioning (5 items).

PedsQL™ Multidimensional Fatigue Scale (PedsQL™ MFS) is an 18-item scale that measures fatigue across three subdomains: general fatigue, sleep/rest fatigue, and cognitive fatigue. PedsQL™ Cancer Module (PedsQL™ CM) is a 27-item module that evaluates symptoms and concerns specific to pediatric oncology patients, covering eight domains: pain, nausea, procedural anxiety, treatment anxiety, worry, cognitive problems, perceived physical appearance, and communication.

Scoring follows a standardized approach. Items are reverse-scored and transformed to a 0– 100 scale, with higher scores indicating better health-related quality of life (HRQoL).

### Statistical analysis

The statistical analysis was performed using the R software version 4.4.0. Descriptive statistics were used to summarize baseline characteristics. Continuous data are expressed as medians (1st quartile, 3rd quartile), while categorical variables are presented as numbers (percentages). We used the Shapiro-Wilk test to assess the distribution of data. Consequently, we used the t-test, Wilcoxon test, or Wilcoxon rank sum test to calculate the differences between the pre- and post-training periods. Statistical significance was set at α=0.05. A mixed regression model analysis was conducted to investigate the relationship between baseline characteristics, training received, and outcomes. Before conducting the analysis, multicollinearity among dependent variables (i.e., baseline characteristics and training details) was calculated. Only covariates with correlation values lower than 0.7 were included in the model, indicating that there was no multicollinearity among the variables. Subsequently, we performed a mixed regression model analysis, considering the Δ values of clinical outcome scales (Post-training – pre-training) as independent variables and baseline characteristics and training information as dependent variables. A backward selection method was adopted to exclude non-significant covariates.

## Results

### Feasibility results

Between May 2021 and June 2023 (25 months), a total of 35 pediatric oncology patients were enrolled in the telemedicine-integrated adapted physical activity program. The recruitment time for the participants was 12 days. Of these, 18 participants completed the full intervention and provided both baseline and post-intervention data. Seventeen participants dropped out prior to study completion.

The reasons for dropout were categorized as follows: 5 due to poor adherence by the child, 5 due to poor adherence by the parents, 5 due to clinical deterioration, and 2 due to death.

Adherence is a key feasibility indicator; in this study, poor adherence was cited as a reason for dropout in some cases. Among completers, adherence rates will be reported to provide insight into the acceptability and practicality of the intervention. The average program adherence rate was 77,08%, calculated as the ratio of completed sessions to scheduled sessions.

**Figure 1:**
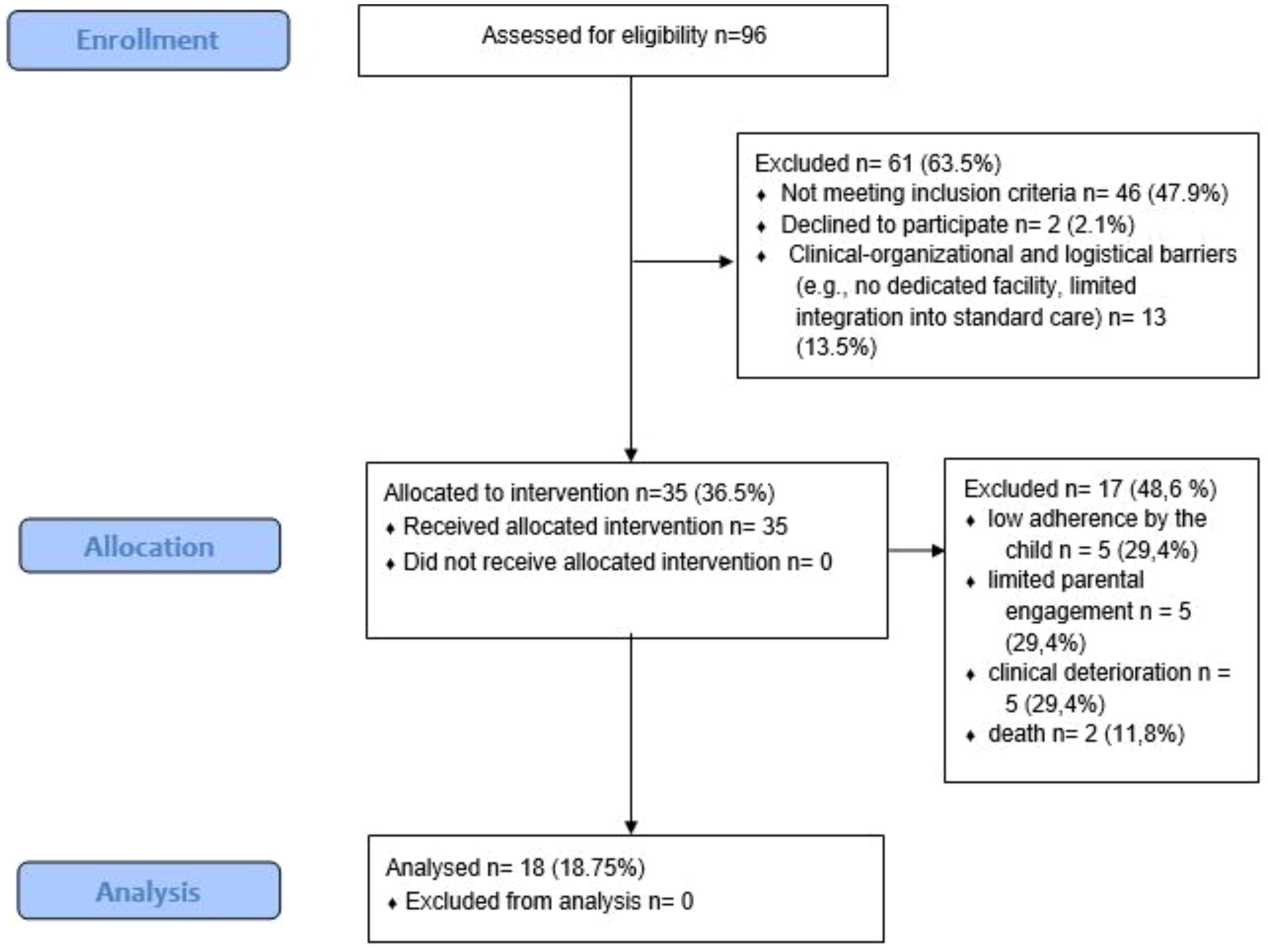
Flow chart showing the total number of children assessed for eligibility, the number included reasons for exclusion, and dropouts with their respective reasons.

A flow diagram (**Figure 1**) will be included to summarize participant flow through the study: 96 individuals were assessed for eligibility, of whom 35 were enrolled. Of these, 18 completed the study and 17 dropped out, with dropout reasons subgrouped accordingly.

The participant characteristics of the enrolled sample are presented in **Table 1**.

**Table 1:** Participant characteristics.

Recruited sample	N = 18
Sex	
Female	8 [44%]
Male	10 [56%]
Diagnosis	
HEMA	10 [56%]
SOLID	8 [44%]
Bone marrow transplant	8 [44%]
Days from diagnosis	100.50 (24.75, 325.00)
Age	12.00 (9.00, 14.00)

Median Interquartile Range(IQR).

Adherence and specific information about the number of sessions performed are reported in **Table 2**.

**Table 2:** Training schedule and adherence.

Recruited sample	N = 18
Trainings sessions executed	55.50 (43.25, 59.75)
Percentage of Adherence to trainings	77.08 (60.07, 82.99)
Face To Face (FTF) trainings	18.50 (10.00, 31.25)
Percentage of FTF trainings	29.55 (19.97, 61.44)
Remote Trainings (RT)	30.50 (19.00, 44.75)
Percentage of RTs	70.45 (38.56, 80.03)

Median Interquartile Range (IQR).

To comprehensively evaluate the feasibility of the intervention, we adopted the structured framework proposed by Cramer et al. (11), which outlines key questions related to recruitment, adherence, acceptability, and protocol implementation in pilot trials. Below, we report our findings according to these feasibility domains.

1. Will I succeed in recruiting enough patients?

Out of 96 pediatric oncology patients assessed for eligibility, 46 did not meet the inclusion criteria. Among the 50 eligible patients, 35 (70%) were enrolled over a 25-month period. These data support the feasibility of recruitment in this population and timeframe.

2. Will the patients be willing to be randomized?

Although the study did not involve randomization, post-intervention feedback indicated that 80% of the enrolled families would be willing to participate in a randomized trial, suggesting good acceptability for future controlled study designs.

3. Can I prevent patients from dropping out of the study early?

Seventeen out of 35 participants (48.6%) withdrew before completing the program. Reasons included low adherence by the child (n=5), limited parental engagement (n=5), clinical deterioration (n=5), and death (n=2). While clinical fragility cannot be modified, dropouts related to adherence may be reduced through the provision of dedicated spaces and improved dissemination of the importance of physical activity in pediatric oncology care.

4. Can I conduct the intervention in accordance with the protocol?

Among the 18 participants who completed the program, adherence to the scheduled training sessions was 77.08%, indicating satisfactory fidelity to the intervention protocol.

5. Can the assessments be implemented as planned or are they too burdensome for patients?

All planned assessments were completed by the participants who finished the program. No dropouts were attributed to the burden of assessments, suggesting that the evaluation procedures were acceptable and manageable.

6. Is the intervention acceptable to patients?

The overall adherence rate of 77.08%, combined with the absence of dropouts directly related to the intervention itself, supports its acceptability. The use of remote delivery may have facilitated integration into family routines, further enhancing its feasibility.

### Secondary results

The comparison of the outcomes considered in the study yielded relevant results for all outcomes related to patient condition (i.e., PedsQL™ GCS, PedsQL™ MFS, PedsQL™ CM, and Chair stand). However, balance (i.e., PBS) and strength (i.e., quadriceps and handgrip strength of both sides) display no remarkable effect. Regarding the parents’ perception outcomes, PedsQL™ MFS parents and PedsQL™ CM parents show a improvement (respectively, p = 0.019 and p = 0.031), while PedsQL™ GCS parents present no improvement (p = 0.067). A comprehensive summary of all comparisons is provided in **Supplementary Table 3a**, located in **Supplementary Material 3**.

**Figure 2:**
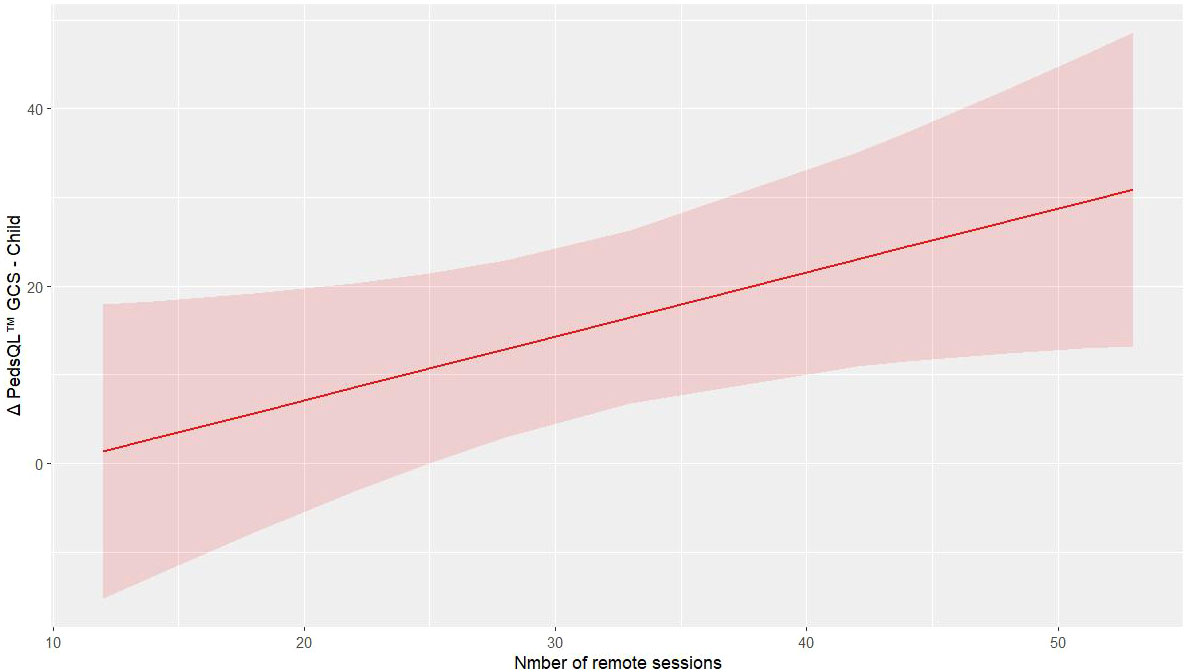
Predictive model of Δ PedsQL™ GCS – Child.

The analysis through mixed regression model analysis indicates that remote training in the PedsQL™ GCS child outcome improves the values of the outcome alongside the increased number of remote sessions (p = 0.027, R2 = 0.235, **Supplementary Table 3b**), **Figure 2** shows the predictive model of PedsQL™ GCS child change concerning the absolute number of remote trainings performed. In contrast, the regression model for the PedsQL™ MFS child provided insufficient evidence for an effect of remote training on the outcome (p = 0.148, R² = 0.116). In the chair sit and reach test, there was no considerable improvement in right and left quadriceps strength in the pre-post comparison. However, remote training appears to play a meaningful role, suggested by the observed trend: p-values of 0.004, 0.020, and 0.003 (respectively, R² = 0.505, 0.359, and 0.887).

**Figure 3:**
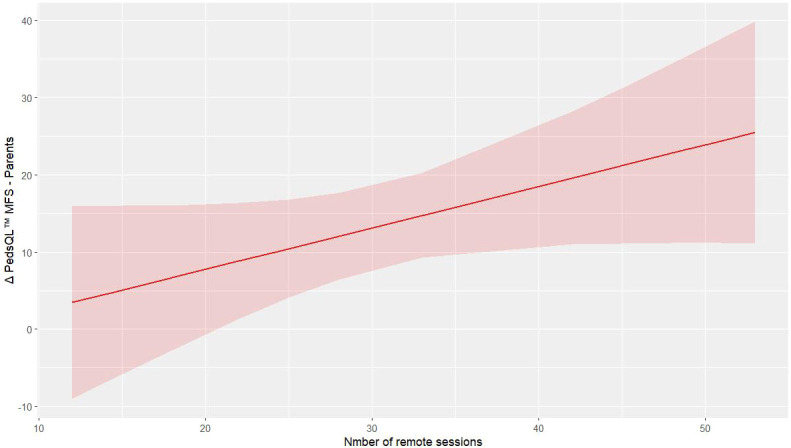
Predictive model of Δ PedsQL™ MFS – Parents.

For the outcomes related to the parents, remote training improves the PedsQL™ MFS score of parents (p = 0.049, R² = 0.718, **Supplementary Table 3c**). **Figure 3** illustrates the predictive model for parents’ improvement in fatigue.

A summary of the regression models for all other outcomes considered is presented in **Supplementary Material 2**.

## Discussion

This study evaluated the feasibility and adherence of a telemedicine-integrated APA program in pediatric oncology patients. Our findings demonstrate that telehealth-based exercise interventions represent a feasible and safe approach. Moreover, our results suggest that an intervention of Adapted Physical Activity can improve quality of life and functional capacity in this vulnerable population, supporting the growing body of literature that emphasizes the importance of exercise interventions in mitigating the long-term sequelae of childhood cancer (15, 16).

The high adherence rate (median 77.08%) observed in our cohort aligns with previous research and further underscores the potential of telehealth-driven approaches to circumvent traditional barriers to exercise participation among pediatric cancer patients (15–17). The main barriers to individual training sessions were related to side effects (e.g., fatigue, nausea), scheduling conflicts due to family or school commitments, and occasional technical issues. Nonetheless, the high adherence observed supports the feasibility of the program and highlights the potential of telemedicine to overcome common barriers to exercise participation.

One of the most compelling results of our intervention was the considerable improvement in HRQoL, as evidenced by the increases in both child- and parent-reported PedsQL™ scores. The observed enhancement in child-reported PedsQL™ GCS scores (p=0.007) is consistent with previous research demonstrating that structured physical activity interventions can positively impact the psychosocial functioning of children undergoing or recovering from cancer treatment (18). The improvement in PedsQL™ CM measures further suggests that our intervention addressed concerns particularly relevant to the oncology experience, including treatment-related symptoms and emotional challenges. Indeed, beyond improving physical health, these programs appear to support emotional well-being and social functioning, components that are often compromised by the disruptive nature of pediatric oncology care (19, 20).

The reduction in fatigue symptoms, as measured by the PedsQL™ MFS Scale (p=0.035), represents another finding. Cancer-related fatigue is considered one of the most pervasive and distressing side effects of childhood cancer, persisting even after the completion of therapy (21). Our results support the growing body of evidence indicating that appropriately prescribed and supervised physical activity can alleviate fatigue in this population by improving cardiorespiratory fitness and enhancing overall endurance (22, 23). Importantly, the improvement in parental perceptions of their children’s fatigue (p=0.019) suggests that these benefits in day- to-day functioning were noted not only in direct patient reports but also observed by caregivers as well.

Regarding the functional outcomes, the remarkable improvement in Chair Stand Test performance (p=0.033) demonstrates enhanced lower limb strength and endurance following the intervention. These gains are particularly relevant given that treatment-related deconditioning commonly impacts mobility and activities of daily living in pediatric cancer patients (24). However, the lack of relevant improvement in isolated quadriceps strength measurements contrasts with these functional gains, suggesting that our intervention may have enhanced neuromuscular coordination and functional movement patterns rather than isolated muscle strength, underscoring the capacity of exercise to enhance broader physical capabilities. This phenomenon has been previously described by Ness et al. (25), who found that childhood cancer survivors often demonstrate functional performance deficits that cannot be attributed solely to decreases in muscle strength.

Furthermore, the regression analysis indicated that remote sessions played an important role in enhancing flexibility and quadriceps outcomes (specifically, the left quadriceps), suggesting that targeted exercise components delivered via telemedicine may be particularly effective for certain muscle groups or physical functions.

The regression analysis also revealed that remote training frequency was positively correlated with improvements in child-reported QoL (p = 0.027, R² = 0.235). This relationship highlights the potential value of telehealth delivery in improving psychological outcomes, potentially by reducing the burden of travel, minimizing disruptions to normal routines, and increasing the accessibility of interventions (20, 26).

In parallel, the improvements observed in parent-reported measures of both cancer-specific QoL (p = 0.031) and fatigue (p = 0.019) indicate that caregivers perceived meaningful changes in their children’s well-being. Parent-proxy reports provide valuable complementary information in pediatric assessment, particularly for younger children or when evaluating observable behaviors (19). The positive correlation between remote training frequency and improvements in parent-reported fatigue scores (p = 0.049, R^2^ = 0.718) suggests that telehealth delivery may have enhanced parental engagement and awareness of their children’s progress.

### Relevance and clinical implications

Several factors may have contributed to the success of our telehealth-integrated approach. First, the personalized nature of the intervention, tailored to each participant’s physical capacity, clinical needs, and psychological condition, likely enhanced both adherence and effectiveness. This aligns with recommendations from Morales et al., who emphasized the importance of individualized exercise prescriptions in pediatric oncology. Second, the supervision provided by qualified professionals ensured that exercises were performed safely and effectively, addressing concerns about the appropriateness of remote delivery for clinical populations (27).

Third, integrating both in-person and remote sessions may have provided an optimal balance, allowing for periodic hands-on assessment and adjustment while maintaining the accessibility benefits of telehealth.

Based on these findings, our study has important implications for clinical practice. The implementation of a telehealth-integrated APA program in pediatric onco-hematology proved to be feasible and further demonstrated that digital health solutions can effectively extend specialized care based on therapeutic exercise and/or rehabilitation interventions beyond traditional settings. By eliminating geographical barriers, this approach may ensure equitable access to APA programs and be particularly valuable in addressing disparities in access to supportive care services, especially for patients residing in rural or underserved regions (17). Additionally, remote supervision enables sustained engagement in exercise regimens during periods when in-person visits may be challenging. The high adherence rates suggest that telehealth delivery may enhance engagement with exercise and rehabilitative programs, potentially leading to improved long-term outcomes, including continuity of care.

Furthermore, in terms of cost-effectiveness, telehealth reduces indirect costs associated with transportation and time off work for caregivers.

Altogether, these advantages highlight the potential for telemedicine to become a standard component of survivorship care plans for pediatric cancer patients.

### Strengths and limitations

A notable strength of this study lies in its blended in-person and remote design, which not only appears to elevate adherence rates but also broadens accessibility for families. Moreover, the tailored nature of the intervention, with adjustments for each child’s clinical and psychological status, is consistent with best-practice guidelines for pediatric exercise prescription (27).

Despite the promising findings that emerged from the study, several limitations must be acknowledged.

Most notably, this was a non-controlled feasibility clinical trial, limiting the ability to draw definitive causal inferences (28). However, the contribution from natural recovery processes is likely to be minimal in this context.

The sample size was relatively small (N = 18), which limits the generalizability of the findings and may have reduced the statistical power to detect differences in some outcomes. Additionally, the heterogeneity of our samples in terms of diagnoses, treatment protocols, and time since diagnosis may introduce confounding effects.

Lastly, while substantial, the six-month duration of the study may not be sufficient to characterize long-term adherence and the effects of the intervention.

### Future directions

Future research should address the limitations above through larger scale, randomized controlled trials (with adequate subgrouping) and extended follow-up periods to assess whether initial improvements are sustained. Studies comparing different telehealth delivery models (e.g., synchronous vs. asynchronous, varying frequency and intensity, and different exercise and physical therapy approaches) would help optimize intervention design and maximize benefits across various functional domains. Investigation of potential moderating factors, including diagnosis, treatment intensity, and baseline functional status, would enable more precise targeting of interventions. Ultimately, cost-effectiveness analyses would provide valuable information for healthcare systems considering the implementation of telehealth-based exercise programs.

## Conclusions

In conclusion, this study provides preliminary evidence supporting the feasibility of a telemedicine-based APA program in enhancing QoL and functional performance among pediatric oncology patients. The statistically significant changes observed are promising, although they should be interpreted as supportive rather than definitive proof of effectiveness. The strong relationship between remote training frequency and improvements in several outcomes highlights the potential value of telehealth delivery in extending specialized supportive care to this vulnerable population, reinforcing the crucial role that exercise, and telehealth can play in survivorship care. The findings, thus, support the integration of digital health solutions into long-term survivorship care while highlighting areas for further investigation. More extensive trials and long-term investigations are essential to build upon these findings, ultimately guiding clinicians, researchers, and policymakers in optimizing supportive care for children and adolescents with cancer by integrating digital health solutions services. Indeed, as healthcare systems increasingly adopt telehealth technologies, tailored exercise interventions could play a pivotal role in addressing the complex needs of childhood cancer survivors.

## Data Availability

The original contributions presented in the study are included in the article/**Supplementary Material**. Further inquiries can be directed to the corresponding author.
