# Supplementary material for: Telemedicine-based adapted physical activity programs for pediatric oncology patients in active oncological care: a feasibility study
Source: Front Oncol. 2025 Sep 8;15:1634626. doi: 10.3389/fonc.2025.1634626 (PMC12450653; doi:10.3389/fonc.2025.1634626)
Supplement: Supplementary file 1 [file DataSheet1.pdf]

TIDieR

1. Telemedicine-integrated Adapted Physical Activity (APA) program.
2. Exercise medicine is gaining increasing importance in the healthcare management of oncologic patients. However, the pediatric field remains challenging due to the biopsychosocial condition of the subjects. In this context, it is important to evaluate the feasibility, adherence, and effectiveness of a telemedicine-integrated adapted physical activity (APA) program for improving functional and psychosocial outcomes in pediatric oncology patients.
3. The intervention was carried out through a fitball, elastic bands, dumbbells (1 to 3 Kg), balance disc, balls, cones, obstacles, medball. All interventions were monitored through a pulse oximeter. In a telemedicine session, a PC or tablet was used with a webcam, and the Cisco WebEx platform was used.
4. At the beginning of the intervention, an exercise specialist prepared a short-term program (for each session) and a long-term program (progression throughout the whole APA program). The single session lasted from 30 to 50 minutes according to the subject condition. The session was divided into different activities, usually divided into an initial warm-up (20% of the total session time), strength training (20% of the total session time), balance training (10% of the total session time), aerobic training (30% of the total session time), and cool-down (20% of the total session time). The setting was previously prepared through the sanitization of the material and the hospital bedroom (for face-to-face sessions). Each subject was carefully evaluated by the exercise specialist and sports physician before each session. Feedback was asked to verify physical exertion and perceived training quality at the end of the single sessions.
5. The sessions were delivered by three exercise specialists, with at least 10 years of experience, and the supervision of the sessions was performed by a sports physician with 5 years of experience.
6. Sessions were delivered one-to-one; the mode of delivery was face-to-face or telemedicine.
7. Face-to-face sessions were performed in the subject's hospital bedroom, while telemedicine sessions were carried out in a home setting without obstacles and with at least a 2x2-meter space.
8. A total of 72 sessions were planned (3 sessions per week, for 24 weeks). Each session could last from 30 to 50 minutes. Session intensity could be adapted according to the subject-specific condition.
9. The pre-exercise assessment and the feedback provided at the end of the previous session, combined with the intrasession feedback, were used to personalize the intensity of exercises during the sessions.
10. The only change performed was the session setting, face-to-face or telemedicine, according to the health status of the subjects.
11. Adherence was monitored, and the setting of each session was recorded by the exercise specialists.

12. The general adherence was high (>75% of session attendance). However, participants had to be encouraged to participate in the session through game therapy.
