# Supplementary material for: Telemedicine-based adapted physical activity programs for pediatric oncology patients in active oncological care: a feasibility study
Source: Front Oncol. 2025 Sep 8;15:1634626. doi: 10.3389/fonc.2025.1634626 (PMC12450653; doi:10.3389/fonc.2025.1634626)
Supplement: Supplementary file 2 [file DataSheet2.pdf]

Supplementary material 2

	**Δ CANCER child**		
Predictors	*Estimates*	*CI*	*p*
(Intercept)	9.30	3.83 – 14.76	**<0.001**
Observations	15		
R^2	0.000		

	**Δ FATIGUE child**		
Predictors	*Estimates*	*CI*	*p*
(Intercept)	-2.01	-16.75 – 12.73	0.789
Remote trainings	0.31	-0.11 – 0.74	0.148
Observations	18		
R^2	0.116		

	**Δ chair stand**		
Predictors	*Estimates*	*CI*	*p*
(Intercept)	-1.40	-12.08 – 9.29	0.798
BMT [yes]	-8.99	-14.80 – -3.19	**0.002**
Age	1.07	0.12 – 2.02	**0.027**
Observations	16		
R^2	0.475		

Δ PBS

Predictors	*Estimates*	*CI*	*p*
(Intercept)	0.62	-0.54 – 1.77	0.294
Days from diagnosis	0.00	-0.00 – 0.01	0.054
Observations	15		
R^2	0.223		

	**Δ chair sit and reach rigth**		
Predictors	*Estimates*	*CI*	*p*
(Intercept)	-27.22	-71.65 – 17.21	0.230
Death [yes]	-14.32	2.49 – -31.13	0.095
Days from diagnosis	0.03	0.01 – 0.06	**0.016**
FTF trainings	0.82	-0.05 – 1.70	0.064
Remote trainings	1.00	0.33 – 1.68	**0.004**
Observations	17		
R^2	0.505		

	**Δ chair sit and reach left**		
Predictors	*Estimates*	*CI*	*p*
(Intercept)	-36.14	-71.19 – -1.09	**0.043**
Days from diagnosis	0.02	0.00 – 0.05	**0.044**
FTF trainings	0.53	-0.22 – 1.28	0.164
Remote trainings	0.69	0.11 – 1.28	**0.020**
Observations	17		

R^2	0.359

	Δ right quadriceps strength		
Predictors	*Estimates*	*CI*	*p*
(Intercept)	4.07	-2.28 – 10.42	0.209
Diagnosis [ALL]	-6.51	-12.73 – -0.29	**0.040**
BMT [yes]	-13.52	-23.55 – -3.48	**0.008**
Death [yes]	9.63	0.59 – 18.66	**0.037**
Days from diagnosis	0.02	0.00 – 0.03	**0.012**
FTF trainings	0.18	-0.06 – 0.43	0.147
Observations	12		
R^2	0.586		

	Δ left quadriceps strength		
Predictors	*Estimates*	*CI*	*p*
(Intercept)	-19.15	-34.88 – -3.42	**0.017**
Diagnosis [ALL]	6.58	-11.43 – -1.73	**0.008**
BMT [yes]	21.76	-30.85 – -12.67	**<0.001**
Complications [yes]	5.18	-11.75 – 1.39	0.122
Death [yes]	-12.15	5.82 – 18.49	**<0.001**
Days from diagnosis	0.02	0.01 – 0.03	**<0.001**
Remote trainings	-0.28	-0.47 – -0.09	**0.003**

Observations	12
R^2	0.887

	Δ right handgrip		
Predictors	*Estimates*	*CI*	*p*
(Intercept)	-2.91	-12.45 – 6.63	0.550
BMT [yes]	4.00	-7.06 – -0.94	**0.011**
Complications [yes]	-2.40	-0.65 – 5.45	0.123
Death [yes]	-2.89	-1.31 – 7.09	0.177
Days from diagnosis	0.01	0.00 – 0.01	**0.012**
FTF trainings	0.16	0.00 – 0.31	**0.044**
Remote trainings	0.08	-0.04 – 0.20	0.191
Observations	16		
R^2	0.651		

	Δ left handgrip		
Predictors	*Estimates*	*CI*	*p*
(Intercept)	4.48	-2.50 – 11.47	0.208
Diagnosis [ALL]	2.30	-4.53 – -0.07	**0.043**
BMT [yes]	1.90	4.28 – -0.48	0.117
Complications [yes]	-4.17	1.39 – 6.95	**0.003**
Death [yes]	-4.13	0.80 – 7.47	**0.015**

FTF trainings	0.13	0.01 – 0.25	**0.039**
Remote trainings	0.07	-0.02 – 0.17	0.145
Observations	16		
R^2	0.667		

**Δ PEDS parents**			
Predictors	*Estimates*	*CI*	*p*
(Intercept)	-11.03	-74.47 – 52.41	0.733
Death [yes]	-17.95	-10.29 – 46.18	0.213
Days from diagnosis	0.03	0.00 – 0.07	**0.045**
FTF trainings	1.25	0.09 – 2.41	**0.034**
Remote trainings	0.69	-0.19 – 1.58	0.125
Observations	17		
R^2	0.340		

**Δ CANCER parents**			
Predictors	*Estimates*	*CI*	*p*
(Intercept)	45.74	10.23 – 81.24	**0.012**
BMT [yes]	-9.32	-3.87 – 22.51	0.166
Age	-1.79	-4.01 – 0.44	0.116
Observations	17		
R^2	0.214		
