# Supplementary material for: Telemedicine-based adapted physical activity programs for pediatric oncology patients in active oncological care: a feasibility study
Source: Front Oncol. 2025 Sep 8;15:1634626. doi: 10.3389/fonc.2025.1634626 (PMC12450653; doi:10.3389/fonc.2025.1634626)
Supplement: Supplementary file 3 [file DataSheet3.pdf]

Table SM3.a Pre-post comparison of outcomes.			
Demographic characteristics	***Pre-APA**, N = 18*[1]	***Post-APA**, N = 18*[1]	***p-value***[2]
PedsQL™ GCS - Parents	*60.88 (49.46, 78.29)*	*76.10 (67.50, 88.70)*	*p=0.067*
PedsQL™ GCS - Child	*63.86 (56.13, 76.68)*	*79.45 (69.95, 89.12)*	*p=0.007*
PedsQL™ MFS - Parents	*59.32 (52.10, 73.61)*	*80.55 (66.70, 93.05)*	*p=0.019*
PedsQL™ MFS - Child	*76.65 (62.16, 78.40)*	*81.23 (77.79, 89.93)*	*p=0.035*
PedsQL™ CM - Parents	*68.09 (55.37, 76.85)*	*84.80 (62.00, 88.90)*	*p=0.031*
PedsQL™ CM - Child	*75.00 (66.67, 79.62)*	*88.05 (76.65, 92.83)*	*p=0.019*
Pediatric Balance Scale	*48.00 (45.25, 48.00)*	*48.00 (47.00, 48.00)*	*p=0.64*
Chair stand	*15.00 (12.00, 18.25)*	*20.50 (15.75, 30.00)*	*p=0.033*
Chair sit and reach right	*-11.00 (-27.00, -4.00)*	*-13.50 (-27.00, -2.00)*	*p>0.99*
Chair sit and reach left	*-10.00 (-24.00, -7.00)*	*-11.00 (-22.75, -5.25)*	*p=0.88*
Strength right quadriceps	*18.02 (8.61, 20.40)*	*17.58 (12.62, 22.60)*	*p=0.48*
Strength left quadriceps	*16.44 (11.74, 17.16)*	*15.60 (12.78, 27.19)*	*p=0.44*
Right handgrip strength	*14.68 (10.38, 19.23)*	*18.03 (10.50, 20.83)*	*p=0.62*
Left handgrip strength	*12.97 (10.15, 17.30)*	*16.07 (9.25, 18.58)*	*p=0.57*
[1] Median (IQR)			
[2] Wilcoxon rank sum test; Wilcoxon rank sum exact test			

Table SM3.b Results of the linear regression model of Δ PedsQL™ GCS - Child			
	Δ PedsQL™ GCS - Child		
Predictors	*Estimates*	*CI*	*p*
(Intercept)	-7.32	-29.35 – 14.71	0.515
Remote training	0.72 [1]	0.08 – 1.36	**0.027**
Observations	18		
R^2	0.235		
[1] Slope values			

Table SM3.c Results of the linear regression model of Δ PedsQL™ MFS - Parents			
	Δ PedsQL™ MFS - Parents		
Predictors	*Estimates*	*CI*	*p*
(Intercept)	21.38	-10.77 – 53.54	0.192
Diagnosis	19.80 [1]	8.04 – 31.55	**0.001**
Complication	-35.43 [1]	-49.73 – -21.12	**<0.001**
Age	-2.30 [1]	-4.25 – -0.34	**0.021**

FTF trainings	1.47 [1]	0.72 – 2.22	**<0.001**
Remote trainings	0.54 [1]	0.00 – 1.07	**0.049**
Observations	17		
R^2	0.718		
[1] *Slope values*			
